# Medical Board Discipline of Physicians for Spreading Medical Misinformation

**DOI:** 10.1001/jamanetworkopen.2024.43893

**Published:** 2024-11-12

**Authors:** Richard S. Saver

**Affiliations:** 1 University of North Carolina School of Law, Chapel Hill; 2 University of North Carolina School of Medicine, Chapel Hill; 3 University of North Carolina Gillings School of Global Public Health, Chapel Hill

## Abstract

**Importance:**

False medical information disseminated dangerously during the COVID-19 pandemic, with certain physicians playing a surprisingly prominent role. Medical boards engendered widespread criticism for not imposing forceful sanctions, but considerable uncertainty remains about how the professional licensure system regulates physician-spread misinformation.

**Objective:**

To compare the level of professional discipline of physicians for spreading medical misinformation relative to discipline for other offenses.

**Design, Setting, and Participants:**

This cross-sectional study analyzed and coded publicly reported medical board disciplinary actions in the 5 most populous US states. The analysis included data from January 1, 2020, through May 30, 2023, for California, Florida, New York, and Pennsylvania and from January 1, 2020, through March 30, 2022, for Texas.

**Main Outcomes and Measures:**

Medical board disciplinary proceedings that resulted in some form of sanction were analyzed. Codes were assigned for the different types of offenses relied on by medical boards for imposing physician discipline.

**Results:**

Among 3128 medical board disciplinary proceedings in the 5 most populous states, spreading misinformation to the community was the least common reason for medical board discipline of physicians (6 [0.1%] of all identified offenses). Two reasons tied for third least common: patient-directed misinformation (21 [0.3%]) and inappropriate advertising or patient solicitation (21 [0.3%]). The frequency of misinformation conduct was exponentially lower than more common reasons for discipline, such as physician negligence (1911 [28.7%]), problematic record-keeping (990 [14.9%]), and inappropriate prescribing (901 [13.5%]). Patient-directed misinformation provided a basis for discipline 3 times as often as spreading misinformation to the community. The frequency of disciplinary actions for any reasons related to COVID-19 care, even if not about misinformation, was also quite low (10 [0.2%]). Sanctions in misinformation actions tended to be relatively light.

**Conclusions and Relevance:**

The frequency of discipline for physician-spread misinformation observed in this cross-sectional study was quite low despite increased salience and medical board warnings since the start of the COVID-19 pandemic about the dangers of physicians spreading falsehoods. These findings suggest that there is a serious disconnect between regulatory guidance and enforcement and that medical boards relied on spreading misinformation to patients as a reason for discipline 3 times more frequently than disseminating falsehoods to the public. These results shed light on important policy concerns about professional licensure, including why, under current patient-centered frameworks, this form of regulation may be particularly ill-suited to address medical misinformation.

## Key Points

**Question:**

How frequently do medical boards discipline physicians for spreading medical misinformation relative to discipline for other professional misconduct?

**Findings:**

In this cross-sectional study of 3128 medical board disciplinary proceedings involving physicians, spreading misinformation to the community was the least common reason for medical board discipline (<1% of all identified offenses). Patient-directed misinformation and inappropriate advertising or patient solicitation were tied as the third least common reasons (<1%); misinformation conduct was exponentially less common than other reasons for discipline, such as physician negligence (29%).

**Meaning:**

Extremely low rates of disciplinary activity for misinformation conduct were observed in this study despite increased salience and medical board warnings since the start of the COVID-19 pandemic about the dangers of physicians spreading falsehoods; these findings suggest a serious disconnect between regulatory guidance and enforcement and call into question the suitability of licensure regulation for combatting physician-spread misinformation.

## Teaser

This cross-sectional study examines the frequency of medical board discipline of US physicians for spreading medical misinformation relative to discipline for other offenses.

## Introduction

False medical information disseminated widely during the COVID-19 pandemic.^1^ Surprisingly, some physicians played a notable role, ranging from discrediting vaccination to promoting ineffective treatments.^2,3,4^ With the increased salience about the dangers of medical misinformation, many commentators called for medical boards to crack down on physicians peddling falsehoods.^5,6^ Reportedly few disciplinary actions resulted, leading to widespread criticism.^7,8^ Yet considerable uncertainty remains about how the licensure system polices misinformation.

The Federation of State Medical Boards (FSMB), the umbrella organization for the state licensing agencies, disclosed in 2021 that 21% of member boards had taken adverse action against physicians for spreading misinformation, but the particular offenses and discipline rates were not revealed.^2^ Media reports have suggested low rates of discipline, but these examinations have been ad hoc in the identification of actions for review.^9,10^ Even more importantly, these prior investigations did not evaluate discipline for spreading misinformation relative to discipline for other misconduct. Such comparison is necessary to account for discretionary enforcement decisions as well as any pandemic-related interruptions in licensure regulation.

This study aimed to fill some of the information gaps and to track how medical boards have policed medical misinformation alongside other offenses. Publicly reported disciplinary actions by state medical boards in the 5 most populous US states (California, Texas, Florida, New York, and Pennsylvania) were analyzed and coded to shed light on important policy concerns within the system for professional regulation.

## Methods

Because this cross-sectional study used publicly available data, it was exempt from human participant review and informed consent under 45 CFR 46.104(d)(4)(i). The study followed the Strengthening the Reporting of Observational Studies in Epidemiology (STROBE) reporting guideline.

### Data Sources and Coding Procedures

The project dataset included all publicly reported licensure actions against physicians in the 5 most populous US states: California, Texas, Florida, New York, and Pennsylvania. Each state’s medical board maintains a website linking to disciplinary proceedings against physicians.^11,12,13,14,15^ These websites can be searched for disciplinary actions by physician name, license number, or date, with links to the underlying legal records such as statements of charges and medical board opinions. Each state’s website was queried for all proceedings involving physicians in the applicable date ranges. The start date was January 1, 2020, to coincide with updated annual reporting by the medical boards and the first month when considerable attention in the US turned to COVID-19.^16^ The end date selected was May 30, 2023, based on the most recent data apparently available when the analysis commenced. This end date (May 30, 2023) remained for all 5 states except for Texas. Due to incomplete files, not all Texas medical board actions after March 30, 2022 were fully accessible on the agency website or available upon alternative information requests. The dataset for Texas therefore included all publicly reported disciplinary actions for January 1, 2020, through March 30, 2022.

A literature review was conducted for discussion of the most common reasons for licensure discipline.^17,18,19,20^ Based on this research, a list of 11 different codes was developed to capture possible offenses leading to medical board discipline, ranging from alcohol or substance abuse to violation of patient confidentiality (Table). Two additional codes were added with regard to spreading medical misinformation: one for false claims to patients and a second for misinformation to the public. The distinction between patient-directed and public-directed falsehoods is important. Medical boards typically monitor physicians’ clinical activities, yet many incidents that attracted criticism involved false communications to the community, such as social media postings or remarks at town meetings.^21,22,23^ An additional category was added to cover physician interactions with patients concerning COVID-19 but was not related to spreading misinformation (eg, failure to evaluate a patient for COVID-19 symptoms before prescribing asthma medication). Finally, a catch-all other category covered conduct not addressed by the previous categories.

**Table.** Reasons for Discipline by Medical Boards

Code	Definition
AD	Advertising or patient solicitation
ASA	Alcohol or substance abuse
CF	Patient confidentiality
CME	Continuing medical education
CR	Criminal activity
C19	Physician–patient interactions related to COVID-19 (but not misinformation)
FR	Fraud unrelated to spreading misinformation
IMP	Physical or mental impairment (unrelated to alcohol or substance abuse)
MMC	Spreading medical misinformation beyond patients to the community or public
MMP	Spreading medical misinformation to patients (ie, under treatment)
N	Negligence, gross negligence, or incompetence in patient care
P	Inappropriate prescribing, including of controlled substances, which includes inadequate documentation for controlled substance prescribing, diversion, or sale
RK	Record-keeping (unrelated to record-keeping for prescribing)
SBM	Sexual or boundary misconduct with patients
Other	Actions taken that did not fit into the previous categories

The dataset was compiled by analyzing and coding all of the reported proceedings involving physicians leading to some sanction. Research assistants with legal training (supervised by R.S.S.) assigned codes reflecting the reasons for discipline in each proceeding. They read all documents available in a particular action, including initial charges, memoranda, and opinions, before coding because often initial allegations covered a broader range of conduct compared with narrower findings and authorities cited in the final disposition. Frequently, multiple codes were assigned to each action because often a medical board imposed discipline in a single proceeding for more than 1 reason.

### Statistical Analysis

Disciplinary activity was assessed by comparing (1) the number of licensure actions involving certain offenses with the total number of underlying offenses and (2) the number of licensure actions involving certain offenses to the total number of licensure actions. Results are reported as frequencies. No statistical software was used for data analysis.

## Results

Across the 5 most populous states for the time periods reviewed (California, Texas, Florida, New York, and Pennsylvania), there were 3128 medical board disciplinary proceedings involving physicians. These actions identified 6655 occurrences of the types of disciplinary offenses described in the Table. The leading reasons for physician discipline were identified based on the number of times a particular type of offense provided a basis for medical board action compared with the total number of offenses underlying all of the disciplinary actions.

**Figure 1.**
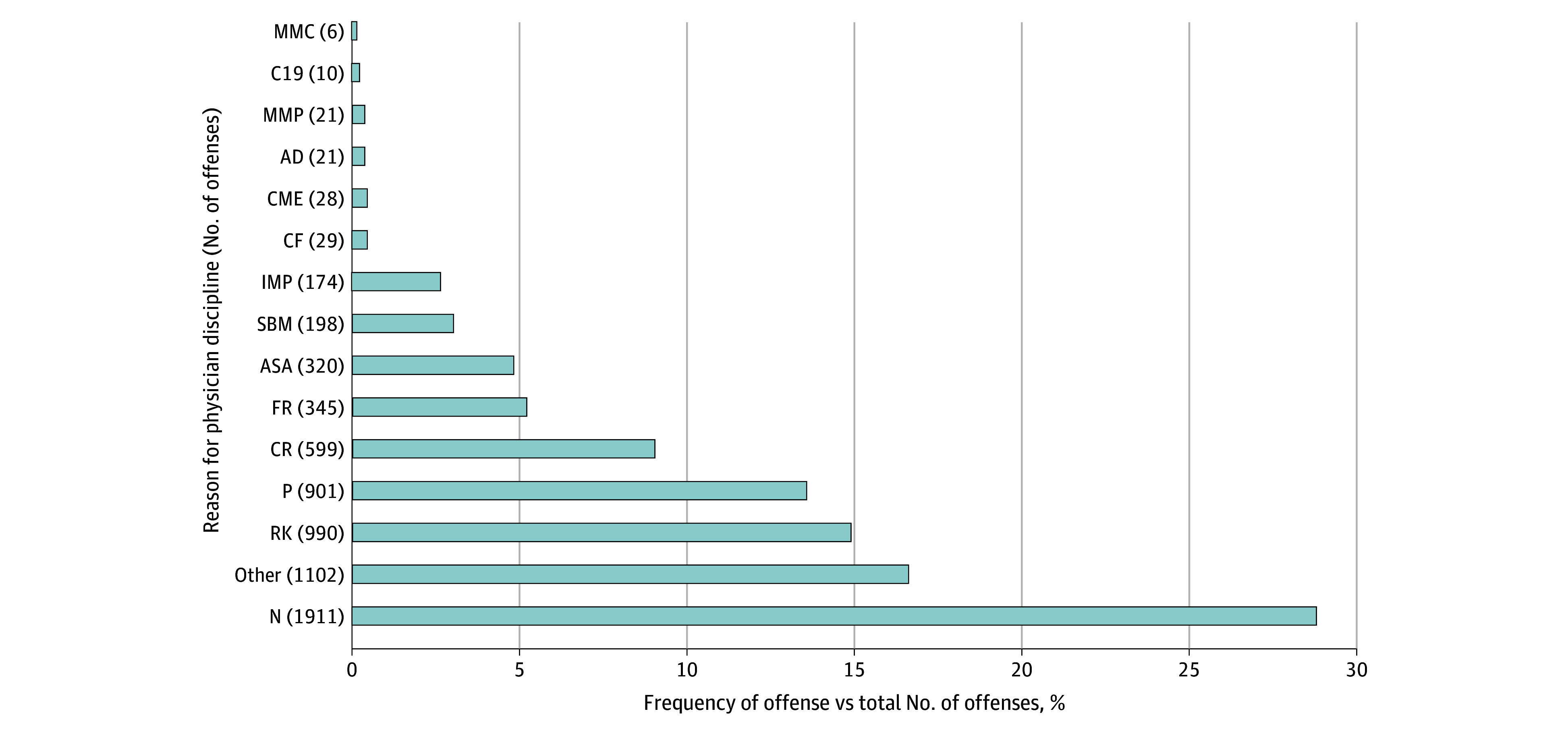
Reasons Underlying Medical Board Discipline in the 5 Most Populous US States, by Frequency of Offense vs Total Offenses Category codes were assigned for the offenses and corresponding codes listed in the Table. Numbers are absolute values vs percentages. Data were from January 1, 2020, through May 30, 2023, for California, Florida, New York, and Pennsylvania and from January 1, 2020, through March 30, 2022, for Texas.

As illustrated in Figure 1, the top 5 reasons for physician discipline across all 5 states were practitioner negligence (1911 [28.7%]), other (1102 [16.6%]), problematic record-keeping (990 [14.9%]), inappropriate prescribing (901 [13.5%]), and criminal activity (599 [9.0%]). Alcohol or substance abuse (320 [4.8%]) and inappropriate relations with patients (198 [3.0%]) were not among the top reasons for physician discipline in this study. Although the catch-all other category was coded for a relatively large number of actions, this was usually because disciplinary action had been automatically and reciprocally triggered, in accord with applicable regulations, when another state’s medical board had disciplined the physician for conduct in that other state. This might occur, for example, when the physician had been convicted of a crime in that other state. Often, the reasons for the other medical board’s initial disciplinary proceeding were not identified.

Misinformation conduct was among the least common reasons for physician discipline. As illustrated in Figure 1, the least common reasons for sanctions were spreading medical misinformation to the public (6 [0.1%]), interactions with patients involving COVID-19 but not misinformation (10 [0.2%]), and, tied for third lowest, spreading medical misinformation directly to patients receiving treatment (21 [0.3%]) and inappropriate advertising or patient solicitation (21 [0.3%]).

**Figure 2.**
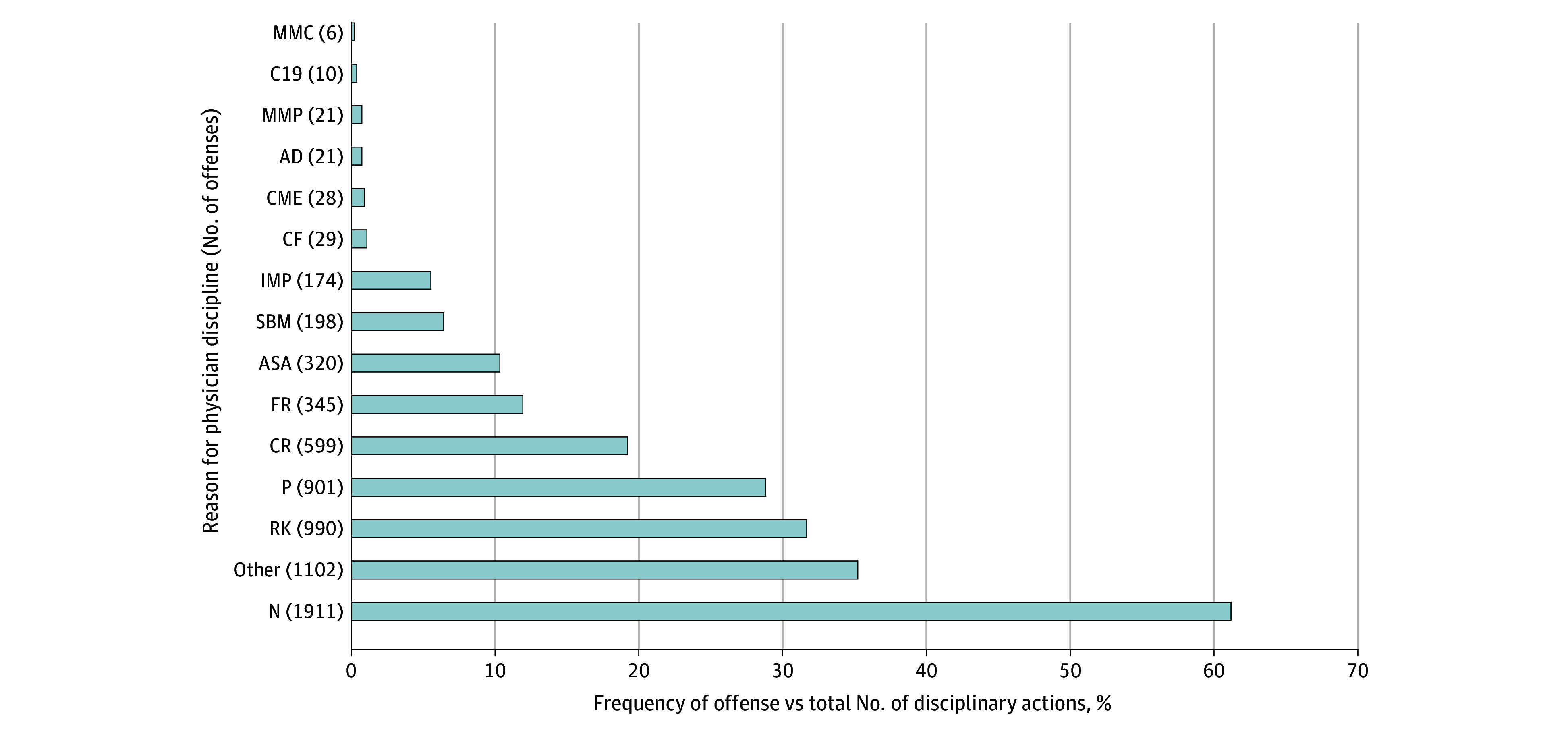
Reasons Underlying Medical Board Discipline in the 5 Most Populous States, by Frequency of Offense vs Total Disciplinary Actions Category codes were assigned for the offenses and corresponding codes listed in the Table. Numbers are absolute values vs percentages. Data were from January 1, 2020, through May 30, 2023, for California, Florida, New York, and Pennsylvania and from January 1, 2020, through March 30, 2022, for Texas.

Another way of evaluating disciplinary activity is to compare the number of licensure actions involving certain offenses to the total number of licensure actions (as opposed to the total number of underlying offenses). Even viewed under this perspective, the occurrence of medical board discipline for spreading misinformation or conduct involving COVID-19 was quite low. As illustrated in Figure 2, the least common offenses were spreading misinformation to the public (6 [0.2%]), conduct involving COVID-19 but not related to misinformation (10 [0.3%]), and, tied for third least common, spreading misinformation directly to patients (21 [0.7%]) and inappropriate advertising or patient solicitation (21 [0.7%]).

Spreading misinformation to patients receiving treatment (21 [0.3%]) formed a basis for discipline 3 times more often than disseminating falsehoods to the public (6 [0.1%]) (Figure 1). Penalties in the misinformation actions tended to be relatively light, such as issuing a public letter of reprimand or placing the physician’s license on probationary status. No disciplinary actions involving misinformation were identified that resulted in licensure revocation, although 1 California physician under investigation voluntarily surrendered their license.

**Figure 3.**
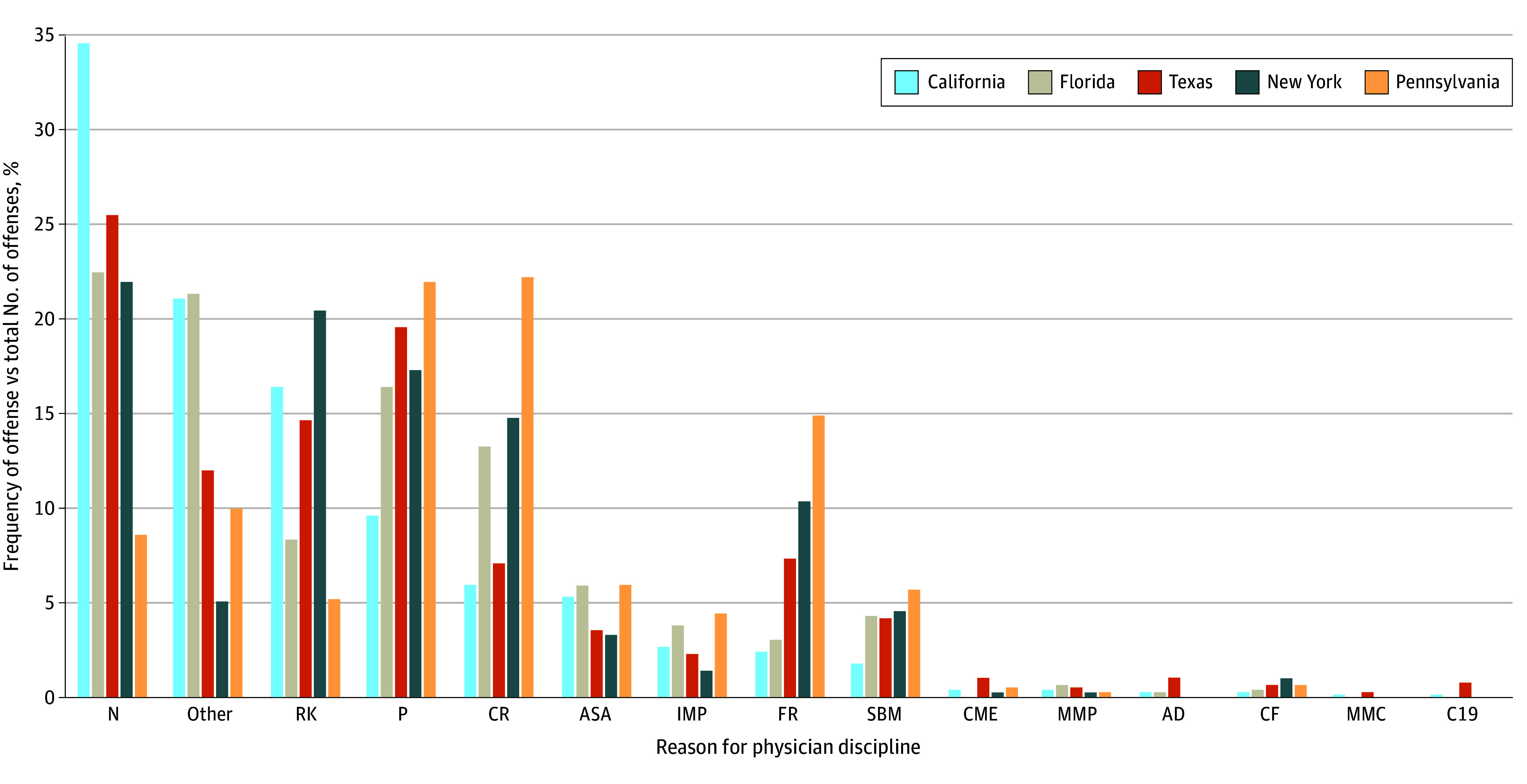
State-Specific Rankings of Reasons for Discipline, by Frequency of Offense vs Total Offenses Underlying All Disciplinary Actions Category codes were assigned for the offenses and corresponding codes listed in the Table. Data were from January 1, 2020, through May 30, 2023, for California, Florida, New York, and Pennsylvania and from January 1, 2020, through March 30, 2022, for Texas.

Although the overall numbers of misinformation offenses were quite low across all 5 states, there were notable differences (Figure 3). For example, no physicians were disciplined in New York for any misinformation offenses, and no physicians were disciplined in Florida or Pennsylvania for spreading misinformation to the public.

Excluding the catch-all other category, negligence and inappropriate prescribing were among the top 4 reasons for physician discipline in all 5 states. Problematic record-keeping and criminal activity were also more highly correlated with disciplinary action across all 5 states (Figure 3).

## Discussion

The purpose of this study was to collect data on medical board discipline since the start of the COVID-19 pandemic to provide a better accounting of how licensure regulation has addressed misinformation relative to other unprofessional conduct. Complicating this analysis is that the prevalence of physicians spreading medical misinformation is extremely difficult to measure. No government entity tracks such data. Complaints still under medical board investigation are not a matter of public record, and complaints that resulted in no formal sanction after the investigation also typically remain confidential.^24^ Moreover, persons exposed to misinformation may not even know it is false or think to report it.

Indirect evidence, however, suggests that physician-spread misinformation is on the rise and has occurred at sufficient levels to present serious harms. The FSMB reported in December 2021 that about two-thirds of medical boards had seen an increase in complaints about physicians spreading COVID-19 misinformation.^2^ In July 2021, the FSMB issued an advisory that physicians disseminating COVID-19 vaccine misinformation risked disciplinary action.^25^ Concerns have been raised that physician-spread misinformation is more prevalent and potent in the social media era because false claims transmit further and more rapidly than in the past.^26^ A 2022 FSMB report noted that because misinformation can go viral online, medical boards “can expect to receive complaints about misinformation … with increasing frequency.”^27^ Other research suggests that certain physicians have acted as misinformation superspreaders. The Center for Countering Digital Hate analyzed social media platforms and identified 12 individuals who accounted for 65% of all antivaccine content during the measurement period in 2021.^28^ Among the so-called disinformation dozen were at least 4 physicians.^28^

The results of this study suggest that limited discipline of physicians for spreading medical misinformation occurred. For the time periods measured and across all 5 states, spreading misinformation occurred as a reason in less than 1% of all identified instances of disciplinary offenses. The least commonly occurring reason for imposing sanctions was spreading misinformation to the community (0.1% of all identified offenses) (Figure 1). Spreading medical misinformation to patients and inappropriate advertising or patient solicitation tied for the third least common reason (0.3% of all offenses). These occurrences were exponentially lower than the more common reasons for discipline, such as practitioner negligence (28.7%). The low discipline for misinformation conduct extended beyond pandemic-related claims because the dataset contained actions for falsehoods about any medical issue, not just COVID-19.

These low rates of discipline occurred despite the increased salience and medical board warnings during this same time frame about physicians spreading falsehoods. These findings suggest that there appears to be a serious disconnect between regulators’ stated intentions about policing misinformation and actual enforcement. A possible reason is the difficulty in defining medical misinformation. No uniform regulatory definition for the term *medical misinformation* exists. Whether viewed as contrary to scientific consensus or the best available evidence, it remains unclear whether disputed communications should be measured against peer-reviewed literature, professional association guidance, or other metrics. Proving that a communication is false can be especially difficult in contexts involving limited evidence, such as newly emerging disease threats.

Relatedly, medical boards face constraints under constitutional law. Licensed physicians have free speech rights, and the First Amendment to the US Constitution protects even false communications. But medical boards can act in a manner that incidentally burdens speech as part of regulating professional conduct. The boundaries between protected speech and speech permissibly sanctioned remain unclear. A 2022 California law clarified the authority of medical boards to discipline physicians for spreading COVID-19 misinformation.^29^ However, federal district courts reached differing results after reviewing legal challenges, with one court enjoining the law as unconstitutionally vague under the Due Process Clause of the Fourteenth Amendment.^30^ California lawmakers repealed the statute, although notably after the time periods reviewed in this study.^31^

The findings of this study also suggest deeper problems, raising doubt as to whether medical boards are institutionally suited to police medical misinformation. Medical boards face substantial resource constraints and suffer from chronic underfunding.^32^ Commentators theorize that as a result, medical boards prioritize more objective, easier-to-prove reasons for discipline, such as a physician’s parallel criminal conviction.^33^ In one noteworthy case, Simone Gold, MD, JD, founder of the controversial antivaccine group America’s Frontline Doctors, widely promoted discredited COVID-19 treatments. She also pled guilty to a federal misdemeanor for participating in the US Capitol riot on January 6, 2021. The plea automatically led California’s medical board to place Dr Gold’s license on inactive status.^34^ It was only the Capitol riot conduct that triggered any sanction despite her very public work in spreading medical misinformation.

The theorized prioritization tracks strongest with the main observations here that misinformation offenses were among the least common reasons for discipline. Misinformation is substantially more burdensome and time-consuming for medical boards to investigate, for example, involving monitoring of social media and community meetings—surveillance activity that is unfamiliar territory.

Largely reactive, inconsistent uptake procedures may additionally explain the limited discipline for misinformation observed in this study. Most licensure investigations are complaint driven, with the majority of complaints arising from patients and their families.^35^ Although patients may know to complain to medical boards when they experience physical injury, they are less likely to understand, because of expertise asymmetries and trust placed in the healing professional, when the physician has exposed them to medical misinformation.

Problems with institutional resilience and independence likely also played a role. Debates about medical falsehoods have become conflated with sharp political disagreements over balancing individual freedoms and governmental powers. For example, Tennessee lawmakers threated to dismantle the state medical board after it issued guidance suggesting that physicians could face discipline for spreading COVID-19 misinformation.^36^ Earlier analyses have observed decreasing discipline by medical boards when their state legislatures became more politically conservative.^37^ However, it may be too simple to align enforcement along such stark political lines.^38^ Nonetheless, the findings of this study suggest that medical boards, which are not typically structured as independent agencies, lack sufficient institutional strength to take on the particularly volatile issue of medical misinformation.

In this study, spreading misinformation to patients formed a basis for discipline 3 times more frequently than disseminating falsehoods to the public (21 [0.3%] vs 6 [0.1%]) (Figure 1). Possible reasons include jurisdictional concerns. A physician’s public communications, not otherwise connected to care for a particular patient, may be considered at best pseudo-professional advice as opposed to practicing medicine within the meaning of a state’s Medical Practice Act. Indeed, considerable confusion exists about the societal role and professional obligations that private physicians undertake when communicating to nonpatients.

Relatedly, when medical boards have to prioritize investigations, the harms arising from misinformation to the community may seem too diffuse and predicated on only an indirect causal connection to the physician’s actions compared to falsehoods with patients. The results of this study suggest far greater medical board activity concerning patient-facing offenses. The top reasons for discipline included general negligence (28.7%), problematic record-keeping (14.9%), and inappropriate prescribing (13.5%) (apart from the catch-all other category) (Figure 1). Medical boards’ seeming preference for regulating patient-facing conduct can lead to inefficient, poor policy, however, because spreading misinformation publicly may inflict wider damage.

Another notable observation was the low rates of discipline in this study for any physician conduct involving COVID-19, such as failure to wear a mask during clinic visits. Offenses involving COVID-19, but not otherwise about misinformation, represented 0.2% of all instances of disciplinary offenses identified (Figure 1). This finding likely reflects the long-standing divide between medicine and public health. Physician licensure regulation has traditionally focused on monitoring the practice of medicine, not public health control measures. Also, many legislatures minimized physicians’ tort liability for treating patients with COVID-19, fearing physicians would otherwise avoid pandemic care obligations.^39^ Medical boards may have been similarly wary of robust licensure discipline overdeterring physicians.

Previous research and commentary suggest that the majority of serious medical board actions involve drug and alcohol impairment, sexual relations with patients, jeopardizing patient care, or criminal activity.^33,40,41^ Yet alcohol or substance abuse (4.8%) and inappropriate relations with patients (3.0%) were not among the top reasons for physician discipline in this study (Figure 1). Perhaps the pandemic affected medical board operations in a manner that skewed attention away from these offenses. In any event, and more consistent with previous studies, the results here suggested that practitioner negligence (28.7%), inappropriate prescribing (13.5%), and criminal activity (9.0%) were among the top 5 reasons for physician discipline.

### Limitations

There are several limitations to this study. First, the data reported by medical boards addressed only actual licensure sanctions imposed after some form of administrative adjudication concluded. As previously discussed, many complaints to medical boards remain confidential.^24^ Further, informal nudging of physicians by medical boards is also not publicly reported. Thus, the dataset may not show the full picture of what medical boards were learning about physician-spread misinformation and how they responded.

Second, related to the confidentiality of complaints, it remains unknown whether physicians spread misinformation more or less frequently than committing other misconduct, such as violating patient confidentiality. Low levels of discipline for misinformation might simply reflect how rarely the underlying behavior occurred. Nonetheless, tracking the frequency of disciplinary proceedings still is a useful proxy for assessing where medical boards were choosing to concentrate their regulatory firepower. The low number of misinformation sanctions in this study suggests troubling regulatory inaction because it occurred against a backdrop of apparently increasing complaints to medical boards about misinformation, heightened salience about the problem, and medical board guidance and research indicating serious concerns.^2,25,27,28^

Third, disciplinary activity between states can be expected to vary based on resources and investigative tools available. Another possible source of variation is the differing political environments. For example, medical boards in more politically liberal states might be expected to act more vigorously in combatting medical misinformation contrary to the views of federal public health authorities than their counterparts in more politically conservative states. As it turns out, the 5 states reviewed in this study included a mix of conservative and liberal states, so there was cross-sampling.

Fourth, a possible time lag exists. Some misinformation conduct that occurred after the start of the pandemic may show up in later cycles of medical board activity. However, numerous disciplinary actions were observed for non-misinformation conduct that likewise started after the pandemic. This suggests that medical boards were sufficiently active in pursuing new investigations.

## Conclusions

In this cross-sectional study of medical board licensure actions across the 5 most populous US states, misinformation conduct was among the least common reasons for physician discipline. In fact, misinformation conduct was exponentially less common than offenses such as physician negligence. Spreading falsehoods to patients formed a basis for discipline 3 times as often as disseminating misinformation to the community, even though community-directed misinformation may pose greater harm overall. These findings shed light on important concerns within the system for professional regulation, including which offenses medical boards seem to prioritize and possible trade-offs in exercising enforcement discretion. Moreover, the study results have serious policy implications, suggesting that the professional licensure system under current patient-centered frameworks may be institutionally ill-suited to combat the diffuse, intractable, and largely public health–related harms arising from physician-spread misinformation.

## Notes

Supplement.
Data Sharing Statement
