# Supplementary material for: Medical Board Discipline of Physicians for Spreading Medical Misinformation
Source: JAMA Netw Open. 2024 Nov 12;7(11):e2443893. doi: 10.1001/jamanetworkopen.2024.43893 (PMC11558475; doi:10.1001/jamanetworkopen.2024.43893)
Supplement: Supplement. — Data Sharing Statement [file jamanetwopen-e2443893-s001.pdf]

Data Sharing Statement

Saver. Medical Board Discipline of Physicians for Spreading Medical Misinformation. *JAMA Netw Open*. Published November 12, 2024. doi:10.1001/jamanetworkopen.2024.43893

Data
Data available: Yes
Data types: Other (please specify)
Additional Information: Will make available medical board proceedings by name and case file numbers in the dataset and associated codes assigned to each proceeding.
How to access data: Data available upon request to author at saver@email.unc.edu
When available: With publication

Supporting Documents
Document types: None

Additional Information
Who can access the data: Researchers requesting the data.
Types of analyses: For research-related purposes.
Mechanisms of data availability: Data available upon request and identification of research-related reason.
